# Case report: Atlantoaxial instability and subluxation in a dog with Ehlers–Danlos syndrome

**DOI:** 10.3389/fvets.2023.1234995

**Published:** 2023-08-04

**Authors:** Simon Choi, Louise Sullivan, Sam Long

**Affiliations:** ^1^Department of Neurology and Neurosurgery, Veterinary Referral Hospital, Dandenong, VIC, Australia; ^2^Department of Veterinary Pathology, Queensland Medical Laboratory, Murarrie, QLD, Australia

**Keywords:** collagenopathy, connective tissue, Ehlers–Danlos, atlantoaxial instability, surgery, congenital

## Abstract

Ehlers–Danlos syndrome is a rare, heritable connective tissue disorder characterized by soft, hyperextensible skin, joint hypermobility, and tissue fragility, the severity of which can range from mild to severe. A 9-month-old male entire miniature Dachshund was presented following peracute tetraparesis. Neurological examination was suggestive of intracranial vestibular disease or high cervical myelopathy. MRI revealed atlantoaxial instability and subluxation, resulting in marked spinal cord compression at C1–C2, which was surgically stabilized. On discharge from the hospital, skin fragility was noted as the result of skin tearing during tape removal. A piece of full-thickness antebrachial skin was submitted for histopathology which showed changes consistent with Ehlers–Danlos syndrome. This case report describes the first case of atlantoaxial instability and subluxation in a dog as the result of a confirmed underlying collagenopathy.

## Introduction

Ehlers–Danlos syndrome (EDS) encompasses a heterogeneous group of rare heritable, congenital connective tissue disorders of varying severity, which are caused by defects in collagen synthesis or assembly and clinically affect the tegmental, musculoskeletal, gastrointestinal, and cardiovascular systems (1, 2). In relation to the integument, defective collagen synthesis results in variable degrees of skin and tissue fragility. In humans, primary clinical manifestations include skin hyperextensibility, easy or spontaneous cutaneous lacerations, skin and vessel fragility, dystrophic scarring, and much less commonly, joint laxity and dislocation (1, 3–6). Connective tissue disorders with resemblance to EDS in humans have been reported in multiple animal species, including dogs, cats, horses, cattle, and sheep (7–10). In dogs, the condition has been described in many breeds, including the Dachshund (11). An autosomal dominant mode of inheritance has been described in dogs and cats (1, 12, 13), along with a report of *de novo* mutations in the COL5A1 gene, suggesting that spontaneous mutations are also possible (14). An autosomal-recessive mode of inheritance has previously been hypothesized in a crossbred female dog with EDS (14). Previous reports of dogs with collagenopathies resembling EDS have shared the characteristic clinical signs of skin hyperextensibility and laxity, bruising or bleeding following minor trauma and dystrophic scars. Other less commonly reported signs in dogs and cats include joint laxity and periodontitis, spontaneous rupture of a large artery, diaphragmatic and perineal hernia, and ocular abnormalities (1, 15–17).

Atlantoaxial instability and subluxation (AAIS) secondary to ligamentous laxity has been reported to be a potential complication for humans with EDS, and the development of spinal instability has been increasingly appreciated among clinicians (6, 18). Currently, there are no reports of neurological manifestations secondary to EDS in dogs. This case report describes the diagnosis and treatment of atlantoaxial subluxation in a dog with Ehlers–Danlos syndrome.

## Case presentation

**Figure 1:**
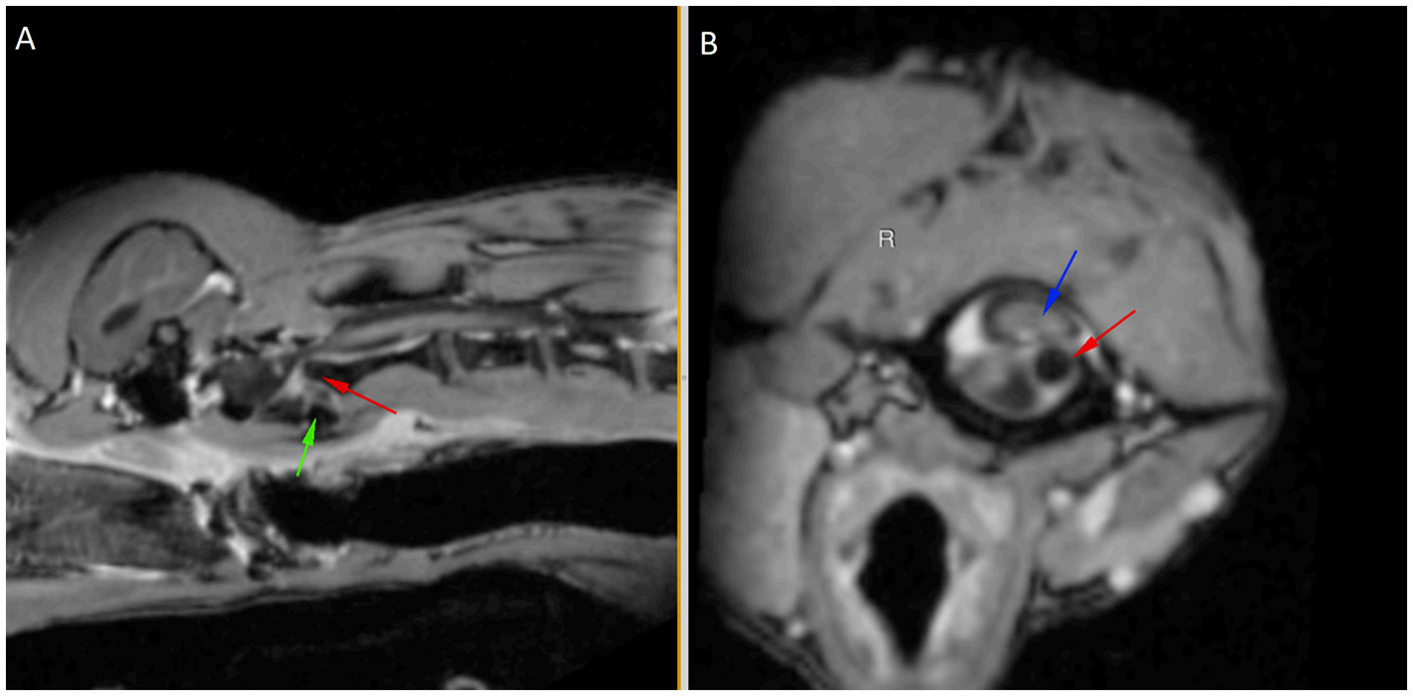
Post-contrast T1-weighted sagittal **(A)** and transverse **(B)** images aligned with the C2 axis to show left and dorsal displacement of the dens (red arrow) in relation to C1 (green arrow shows ventral margin), causing marked cord compression (blue arrow).

A 9-month-old male entire miniature Dachshund presented to the emergency department following peracute tetraparesis after being groomed. Neurological examination revealed normal mentation with non-ambulatory tetraparesis, right-sided head turn, and positional ventrolateral strabismus of the left eye. An intracranial lesion involving the central vestibular system or high cervical myelopathy was suspected. Magnetic resonance imaging (MRI) of the brain and cervical spine was performed using 3.0T Siemens MAGNETOM Skyra (Siemens Healthcare GmbH). Sequences of the brain included sagittal and transverse T2-weighted (T2W) images in 2 mm slices, diffusion-weighted imaging (DWI) in 3 mm slices, apparent diffusion coefficient (ADC) in 3 mm slices, and T1-weighted (T1W) pre- and post-contrast images in 1 mm slices. Cervical spine (caudal cerebellum to T1 vertebrae) sequences included sagittal and transverse T2W (2 mm slices), sagittal T1W pre- and post-contrast in 2 mm slices, and transverse T1W sequences (2 mm slices) from C1 to C3. Images were interpreted by a specialist in diagnostic imaging. MRI of the brain was within normal limits with no structural abnormalities identified. In the cervical spine, the dens had a normal shape with abnormal position, demonstrated by left and dorsal displacement of the dens in relation to C1. There was marked compression of the spinal cord at this site with heterogeneous increased T2W intensity of the cord at C1–C2 (Figure 1).

MRI findings were consistent with atlantoaxial instability/subluxation causing marked spinal cord compression. A conforming ventral fiberglass cast was fabricated and placed to provide external coaptation of the cervical spine.

In-house hematology and biochemistry (IDEXX laboratories) had revealed increased alanine transaminase 212 IU/L (range 13–98), and subsequent investigation via paired bile acid tolerance testing revealed elevated pre- (29, range 0–15) and post-prandial (85, range 0–30) bile acids. Computed tomography scan (Siemens Healthcare GmbH) of the thorax, abdomen, and pelvic limbs were performed using 0.8 mm transverse slices, and pre- and post-contrast images were available, which were reviewed by a specialist in diagnostic imaging. No visible portal-systemic vessel communication was identified. All vessels were identified, appeared normal, and the liver was normal in size.

The patient subsequently underwent surgical stabilization and a ventral approach to the cervical spine was made. The sternohyoideus muscle was identified and divided on midline via blunt dissection with preservation of the thyroid vein. The sternothyroid muscle was transected close to the larynx and trachea retracted to the left side. Blunt dissection to the ventral paraspinal muscles was performed, which were divided on the midline to expose the ventral aspect of C1–C2. The muscles were elevated from these bones and the joint capsule was incised and removed to expose the synovial cartilage. The cartilage on the ventrocaudal and ventrocranial aspects of C1 and C2, respectively, was removed with high-speed burr. C2 was noted to be significantly unstable in relation to C1 and was held in place with a towel clamp placed across the vertebral body. A 1.5-mm hole was drilled from the cranioventral aspect of the left side of C2 across the joint and into C1 with an approximately 40° angle from the midline and with a 20° angle relative to the floor of the spinal canal. A 2.0-mm lag hole was drilled into C2 and an 18-mm self-tapping cortical screw was fastened from C2 to C1. A 1.5-mm hole was then drilled into the right side with the same angles, and the procedure was repeated with a 16-mm 2.0 mm cortical screw on that side. Routine closure was performed with 3/0 PDS simple continuous sutures in the paraspinal, sternothyroideus, and sternohyoideus muscles. The subcutaneous tissue was closed with 3/0 PDS simple continuous sutures, and the skin was closed with 5/0 PDS intradermal sutures. Postoperative ventrodorsal and lateral radiographs were performed, which confirmed that the screws were located appropriately.

A dermal slow-release fentanyl patch (equivalent to 3 μg/kg/h) was placed on one hindlimb, and the patient recovered uneventfully. Postoperative analgesia was achieved with a constant rate infusion of fentanyl (3 μg/kg) for the first 12 h together with meloxicam (0.5 mg/kg PO q24h) and gabapentin (10 mg/kg PO q8h). Repeated neurological examination following surgery revealed slight improvement in the voluntary motor function, but the patient remained non-ambulatory.

On the day of discharge, the adhesive tape covering the cephalic vein catheter on the left antebrachium was removed and a piece of full-thickness skin, approximately 1 cm square, unexpectedly became detached and adhered to the adhesive tape. This occurred under normal handling without the use of excessive force. On further re-examination of the patient, excessive joint laxity, particularly involving flexion and extension in the carpal, stifle, and tarsal joints, was noted together with hyperelasticity of the skin along the dorsal trunk.

**Figure 2:**
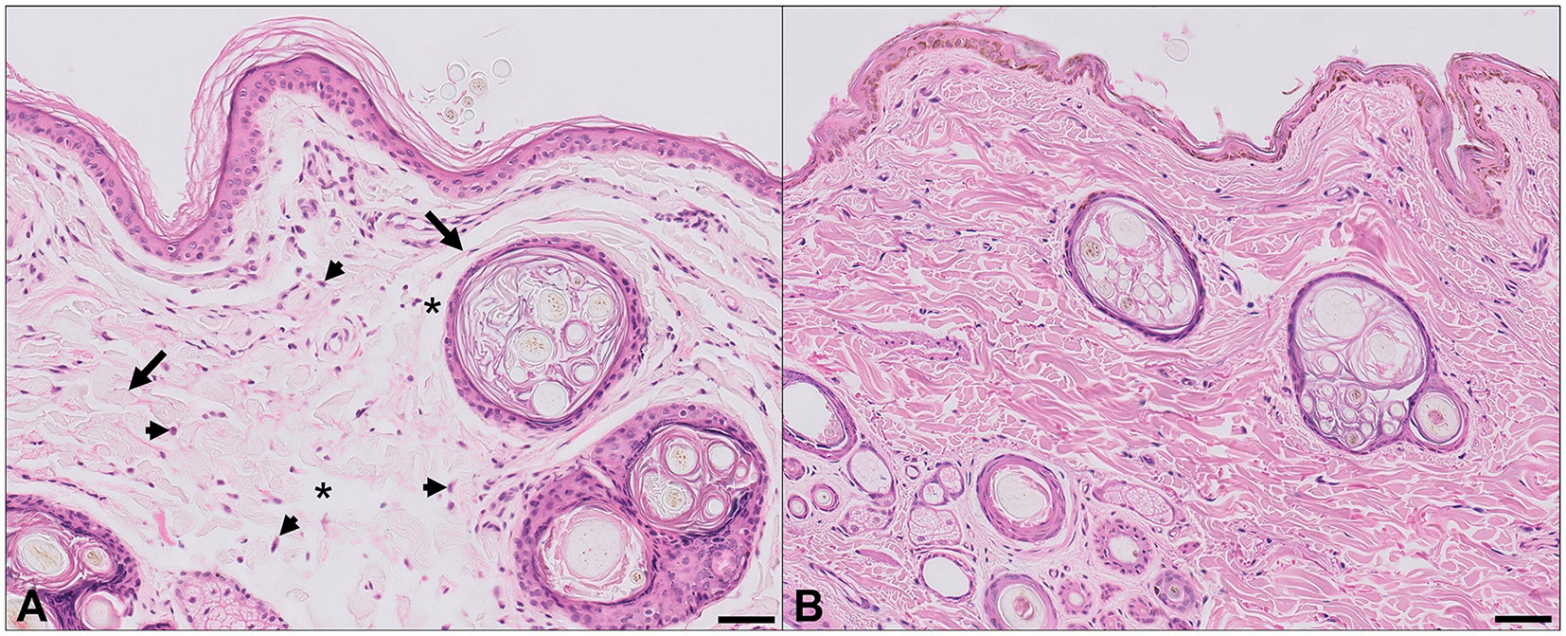
Histopathological findings, antebrachial skin. **(A)** Affected dog. **(B)** Normal dog. Abnormal dermal collagen fibers are irregularly and often loosely distributed, with increased variation in fiber size and width (arrows), and irregular and sometimes large spaces between collagen fibers (asterisks). In some regions there are increased numbers of plump fibroblasts (arrowheads). Hematoxylin and eosin, bar = 50 μm.

The fragment of antebrachial skin that became detached was submitted for histopathology due to the suspicion of abnormal skin fragility and extensibility beyond normal limits. This sample was compared with the samples of antebrachial skin from two control dogs that had no history of dermatological diseases. On routine light microscopic examination of H&E-stained sections, the dermal collagen fibers from this patient were strikingly different from the controls, being irregularly and often loosely oriented, with increased variation in size and shape and increased numbers of dermal fibroblasts (Figure 2). In conjunction with the clinical history, these collagen fiber abnormalities support a collagenopathy consistent with Ehler–Danlos syndrome in the dog discussed in this case. Sections stained with Orcein and Verhoeff-van Gieson stains for elastic fibers did not show clear differences between the patient and controls, and abnormally stained fibers were not identified on the examination of Masson's trichrome-stained sections of the skin.

Follow-up examination at 3 months post-operatively revealed improved neurological function, but the patient remained weakly ambulatory tetraparetic. 13 months postoperatively the patient was readily ambulatory with generalized proprioceptive quality ataxia, and 20 months postoperatively, the patient was ambulatory paraparetic with normal postural reactions in thoracic limbs and delayed in both pelvic limbs. The patient was subsequently lost to follow-up.

## Discussion

To the authors' knowledge, this case report describes the first case of EDS as a presumptive cause of AAIS in a dog. Based on the history, clinical exam, MRI, and dermatopathological findings of the dog, which were supportive of EDS, a collagenopathy directly affecting the supporting ligamentous structures of the atlantoaxial joint is highly suspected. EDS is also termed “cutaneous asthenia” (weak skin) and “dermatosparaxis” (fragile skin). It is mainly characterized by skin fragility, skin hyperextensibility, vascular fragility, and joint hypermobility (4). In humans, a recently revised international EDS classification system describes 13 subtypes which are classified based on clinical signs, inheritance, and biochemical defects (19). However, in veterinary medicine, the classification of collagenopathies is lacking due to few genetic, biochemical, and ultrastructural studies that have been performed (1, 4). Variable extent and severity of tissue and organ involvement can be observed in individual cases due to the diverse molecular and biochemical backgrounds, which ultimately account for the many EDS subgroups and overlapping phenotypes (5). The clinical spectrum can vary from mild skin and joint hyperlaxity to severe physical disability and life-threatening vascular complications (20). The majority of EDS cases in animals are diagnosed based on supportive clinical signs and consistent histopathologic abnormalities of dermal collagen fibers. In humans, EDS is diagnosed based on a combination of clinical signs and testing for known genetic mutations, encoding defective collagen and/or collagen-modifying enzymes (20, 21). Variants in several genes have previously been identified in dogs to aid in subclassifying EDS to corresponding human subtypes. Two dogs have previously been diagnosed with the classical form of EDS via whole-genome sequencing, which revealed *de novo* mutations of *COL5A1*, and a *COL5A2* variant was found in the heterozygous state in Chihuahua (14, 22). A homozygous non-sense variant in *ADAMTS2* was reported to be the likely cause for the dermatosparactic form of canine EDS, while a compound heterozygous missense variant in the *TNXB* gene was found in a mixed-breed dog with EDS (23, 24). Determining the EDS subtype, in this case, would have required fibroblast culture and molecular and biochemical analysis for qualitative and quantitative analyses of collagen; however, these techniques are not readily available in veterinary medicine (16). A genome-wide association study or whole genome sequencing in this dog may have identified the causative genetic variant for the disease, allowing us to distinguish the subtype of canine EDS.

The nervous system is not described as a primary target of EDS, and underlying molecular defects and structural anomalies involving the brain and spinal cord are not common findings in these patients. However, there is an emerging focus in humans with EDS on the wide spectrum of neurological manifestations that have been described and are unexpectedly common and potentially disabling. A recent review discussed the etiology and clinical findings related to neurological and spinal manifestations commonly observed but poorly recognized in people with EDS. The described neurological manifestations included atlantoaxial instability, craniocervical joint instability, segmental kyphosis and instability, and early disc degeneration along with weakness of the epineurium and perineurium surrounding peripheral nerves (6, 22).

Although rare, EDS has been reported to be a cause of atlantoaxial subluxation in people (5, 18), and human EDS patients have presented acutely following either cervical spine dislocation or Os odontoideum (where the dens is separated from the body of the axis) leading to cervical instability and requiring emergency cervical spine fusion (3, 5).

In canines, the majority of atlantoaxial joint stability arises from a series of ligaments that can be divided into two groups: peripheral ligaments which cover the intervertebral space and separate the atlas from the axis and the deep ligaments which are located on the floor of the vertebral canal and connect the dens of the axis to the atlas and the occipital bone. The dorsal atlantoaxial membrane, dorsal atlantoaxial ligament, and ventral atlantoaxial ligament comprise the peripheral ligaments which join the ventral tubercle of the atlas to the ventral crest of the axis. The deep ligaments include the apical ligament of the dens axis, the paired alar ligaments, and the transverse ligament (25). Histologically, alar ligaments consist of wavy collagen fibers and a high proportion of elastic fibers, which provide them with a remarkable elasticity compared with the transverse ligament which constitutes a much denser structure than one of the alar ligaments with highly organized collagen fibers with a much less uniform distribution of elastic fibers (25). Biomechanical evaluation has suggested that the alar ligaments are the most important in providing stabilization of the joint under shear load (26).

Canine AAIS occurs most frequently due to congenital abnormalities and most commonly occurs in young and small toy breeds. Aplasia or hypoplasia of the dens, absence or weakness of supporting ligaments, incomplete ossification of the atlas, block vertebrae, and dorsal angulation of the dens are the most common congenital abnormalities associated with atlantoaxial subluxation. Subluxation can occur in these affected dogs spontaneously or with very mild trauma (4, 27, 28). Traumatic atlantoaxial subluxation from fracture of the dens or ligament rupture can affect any dog; however, it is uncommon and is accompanied by radiographically evident vertebral fractures or evidence of ruptured ligaments on an MRI scan (4, 27). Clinical signs in affected dogs range from cervical hyperesthesia to tetraplegia and, in severe cases, respiratory arrest and death (25).

MRI in this patient did not reveal evidence of absence or tearing of the atlantoaxial ligaments, which further supports excessive laxity as the cause of the atlantoaxial instability and subluxation. The dog in this case report presented following a sudden inability to walk whilst being at the groomers. Dogs with congenital anomalies that predispose them to atlantoaxial subluxation and spinal cord injury may have trauma-related onset of clinical signs. This is likely due to an inability to compensate for the pre-existing instability (29). Minor trauma resulting in acute decompensation of atlantoaxial instability is likely to have occurred in the present case.

The prevalence of AAIS resulting from primary ligamentous disease in dogs is unknown. This is partly due to the lack of preoperative imaging as traditional diagnosis has historically been based on the criteria deduced from CT or survey radiographic studies, which indirectly assess the likelihood of ligamentous rupture (27). Primary ligamentous lesions leading to AAIS have also not been described in the veterinary literature, as no studies have directly assessed the ligaments of these patients. One case report described the absence of the transverse ligament of the atlas on necropsy in a young Shih Tzu with radiographic signs of atlantoaxial subluxation; however, this case exhibited malformation of the dens and atlas (30). In a study by Middleton et al., the apical, alar, transverse, and dorsal atlantoaxial ligaments were identified on MRI in 10 adult small breed canine cadavers, and subjective ligamentous abnormalities were described in three dogs; however, all three dogs had radiographic evidence of hypoplastic or aplastic dens (29).

Reported microscopic changes in animals with EDS include reduced normal dermal thickness and abnormal dermal ± hypodermal collagen fibers (including abnormal size and shape, fragmentation, curling, disarray, and increased space between the fibers). Some cases also have increased numbers of fibroblasts or increased ground substance (1, 31). Histopathologic examination of the skin of young dogs and cats with abnormally fragile and hyperextensible skin usually shows distinct abnormalities in the dermal collagen fibers that are consistent with a diagnosis of EDS (31). In some cases, however, histologic changes in patients with EDS may be subtle or even absent, with a normal microscopic appearance on routine H&E-stained sections reported in affected dogs and cats (12, 32). This variability is presumably due to the existence of multiple syndromes in affected animals, each with unique biochemical aberrations and clinical manifestations. In cases where routine skin biopsy is not diagnostic, further investigations including ultrastructural and biochemical analyses may be necessary (31). A clinical test, the skin extensibility index (SEI), has previously been described to quantitate the extensibility of the skin as a possible indicator for EDS. This involves the lifting of a fold of dorsolumbar skin as far as possible from the spine, and the height of the fold is divided by the body length from the occiput to the tail base and multiplied by 100. The reported normal range is variable from 6 to 15% (1, 12, 33, 34). An SEI was not performed in the present case, although subjectively, skin hyperextensibility was beyond normal limits on clinical examination, which prompted the submission of the skin sample for histopathology, with the subsequent microscopic findings consistent with EDS.

Bloodwork in this patient also demonstrated elevations in ALT and paired bile acid stimulation testing, and CT did not reveal aberrant portal-systemic vessels to account for these biochemical abnormalities. The possibility of other hepatic abnormalities, including portal vein hypoplasia (microvascular dysplasia), cannot be excluded. Human EDS patients with a clinical phenotype involving tissue fragility not only manifest signs externally, such as easy bruising of the skin and impaired wound healing with dystrophic scarring, but can also show fragility within internal organs such as the arteries, lungs, intestines, liver, and spleen. In such cases, a vascular type of EDS is predominantly suspected (35). In the present case, additional diagnostics such as liver biopsy for histopathology may have provided further information regarding the ALT and bile acid elevations; however, these tests were declined by the owner at the time.

## Conclusion

To the best of our knowledge, we describe the first report of a suspected neurological manifestation of Ehlers–Danlos syndrome in a dog. It is postulated that abnormalities in collagen assembly and/or synthesis likely affected the integrity of ligamentous structures surrounding the atlantoaxial joint, subsequently predisposing to atlantoaxial instability and subluxation either spontaneously and/or in the face of minor trauma.

## Data availability statement

The original contributions presented in the study are included in the article/supplementary material, further inquiries can be directed to the corresponding author.

## Ethics statement

Written informed consent was obtained from the participant/patient(s) for the publication of this case report.

## Author contributions

SC and SL were neurology clinicians involved in the clinical patient assessment, imaging work-up, and surgery performed. LS performed the histopathology of the skin that was submitted. SC wrote the first draft of the manuscript. SL and LS wrote sections of the manuscript. All authors contributed to manuscript revision, read, and approved the submitted version.
